# Quantitative ^99m^Tc-Pyrophosphate SPECT/CT evaluation of tafamidis response in a case of transthyretin cardiac amyloidosis

**DOI:** 10.22038/aojnmb.2025.90973.1665

**Published:** 2026

**Authors:** Yoichi Otomi, Hideki Otsuka, Ryosuke Kasai, Tamaki Otani, Noritake Matsuda, Takayuki Ise, Shusuke Yagi, Masataka Sata, Takayoshi Shinya, Masafumi Harada

**Affiliations:** 1 Department of Radiology, Tokushima University Hospital, Tokushima, Japan; 2 Department of Medical Imaging/Nuclear Medicine, Tokushima University Graduate School of Biomedical Sciences, Tokushima, Japan; 3 Advance Radiation Research, Education and Management Center, Tokushima University, Tokushima, Japan; 4 Department of Radiological Technology, Tokushima University Hospital, Tokushima, Japan; 5 Department of Cardiovascular Medicine, Tokushima University Hospital, Tokushima, Japan

**Keywords:** Tafamidis, ^99^mTc-Pyrophosphate SPECT/CT, Quantitative imaging Transthyretin amyloidosis

## Abstract

Transthyretin cardiac amyloidosis (ATTR-CA) is increasingly recognized as a cause of heart failure in elderly patients. Noninvasive diagnosis with technetium-99m pyrophosphate (^99m^Tc-PYP) scintigraphy has become established, but quantitative approaches for therapy monitoring remain under investigation. We present a case of wild-type ATTR-CA in an 82-year-old man treated with tafamidis. Baseline echocardiography showed concentric left ventricular hypertrophy with preserved ejection fraction, impaired global longitudinal strain, and elevated B-type natriuretic peptide (BNP). Planar and SPECT/CT imaging with ^99m^Tc-PYP demonstrated diffuse myocardial uptake (grade 3, H/CL 1.97). Quantitative analysis with GI-BONE software yielded SUV_max_ 4.2, amyloid deposition volume (AmyDV) 122 cm³, and total amyloid uptake (TAU) 313. Endomyocardial biopsy confirmed wild-type ATTR. After 18 months of tafamidis therapy, symptoms persisted with further GLS impairment and BNP elevation, while echocardiographic wall thickness remained increased. In contrast, repeat PYP imaging showed reduced uptake (grade 2, H/CL 1.64) with markedly decreased quantitative indices (SUV_max_ 2.3, AmyDV 2 cm³, TAU 4). This case demonstrates that volumetric indices can capture substantial therapy-related changes, although discordance with functional and biomarker findings highlights the need for integrated assessment. Quantitative ^99m^Tc-PYP SPECT/CT may serve as a promising tool for therapy monitoring in ATTR-CA.

## Introduction

 Transthyretin cardiac amyloidosis (ATTR-CA) is an increasingly recognized cause of heart failure in elderly patients. Misfolded transthyretin proteins form amyloid fibrils that infiltrate the myocardium, leading to restrictive cardiomyopathy, arrhythmias, and poor prognosis. Early diagnosis is essential but often delayed due to overlapping features with other cardiomyopathies. Technetium-99m pyrophosphate (^99m^Tc-PYP) scintigraphy has emerged as a reliable noninvasive diagnostic tool, with visual grading and the heart-to-contralateral (H/CL) ratio validated as key diagnostic methods (1, 2). 

 However, these approaches have limitations in reproducibility and sensitivity to change. Quantitative single-photon emission computed tomography/computed tomography (SPECT/CT) 

now allows volumetric indices to be derived, such as cardiac pyrophosphate volume in prior reports (3), which conceptually parallels indices like amyloid deposition volume (AmyDV) and total amyloid uptake (TAU) used in our study. These methods share a conceptual basis with oncological PET imaging, but their role in monitoring treatment remains underexplored. Tafamidis, a transthyretin stabilizer, improves survival and reduces hospitalizations (4), yet its imaging correlates are not fully defined. We report a case of wild-type ATTR-CA in which tafamidis therapy was associated with marked reduction of quantitative PYP indices, despite persistent symptoms and functional decline. The importance of early and accurate diagnosis, timely therapy initiation, and longitudinal monitoring in ATTR-CA has been emphasized in the recent ESC position statement (5). However, the statement mainly addresses diagnostic and management strategies, and does not mention the usefulness of ^99m^Tc-PYP scintigraphy for monitoring tafamidis-treated patients.

## Case Report

**Figure 1:**
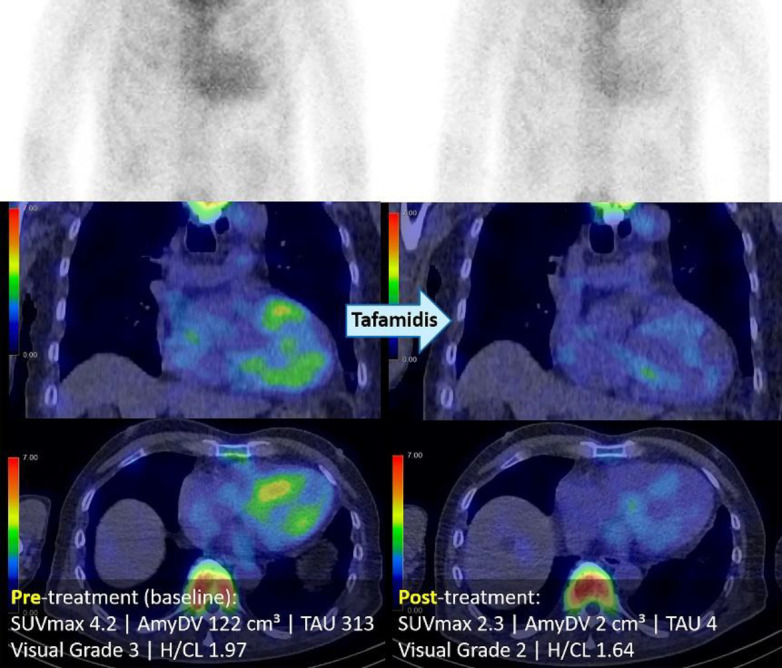
^99m^Tc-PYP planar and SPECT/CT images before (**left**) and after (**right**) tafamidis therapy. Marked visual and quantitative reductions in myocardial uptake are evident post-treatment. Quantitative indices (SUV_max_, AmyDV, TAU, and H/CL ratio) were derived using GI-BONE software (AZE Co., Ltd., Tokyo, Japan)

 An 82-year-old man presented with progressive exertional dyspnea. Baseline echocardiography showed concentric left ventricular hypertrophy with preserved ejection fraction (71%), impaired global longitudinal strain (−10.3%), left atrial enlargement, and mild tricuspid regurgitation. B-type natriuretic peptide (BNP) was elevated at 405.5 pg/ml. Planar and SPECT/CT imaging with ^99m^Tc-PYP (Figure 1, left) demonstrated diffuse myocardial uptake (Perugini grade 3) with an H/CL ratio of 1.97. 

 Acquisition and reconstruction parameters are summarized in Table 1. Quantitative analysis was performed using GI-BONE software (AZE Co., Ltd., Tokyo, Japan). An SUV threshold of 2.1 was established to distinguish myocardial uptake from background activity. These volumetric indices, adapted from oncological PET imaging, were validated in our previous studies on ^99m^Tc-PYP and ^67^Ga-citrate SPECT/CT (6, 7). At baseline, SUV_max_ was 4.2, AmyDV 122 cm³, and TAU 313. Endomyocardial biopsy confirmed transthyretin amyloidosis with Congo red positivity and transthyretin immunostaining. Genetic testing excluded mutations, thereby establishing wild-type ATTR-CA.

 Tafamidis (61 mg/day) was initiated, and the patient was followed for approximately 18 months. Symptoms persisted without improvement. Follow-up echocardiography again demonstrated concentric left ventricular hypertrophy with further impaired GLS (−8.7%) despite preserved ejection fraction (67%). BNP increased to 513.6 pg/ml. In contrast, repeat ^99m^Tc-PYP scintigraphy (Figure 1, right) demonstrated reduced uptake (grade 2, H/CL 1.64). Quantitative SPECT/CT analysis revealed SUV_max_ 2.3, AmyDV 2 cm³, and TAU 4. Thus, imaging showed dramatic improvement while functional and biomarker measures worsened.

**Table 1:** Image Processing Parameters for ^99m^Tc-PYP SPECT/CT

**Parameter**	**Value**
SPECT/CT scanner	Symbia T16 (Siemens)
RI	^99m^Tc-PYP
Collimator	LEHR
keV	140 keV ± 15%
Matrix	128 × 128
Pixel size	3.3 mm
Image processing	Continuous mode
Rotation	180°
Collection time	30 s × 30
Attenuation correction	CTAC

## Discussion

 This case illustrates the potential and limitations of quantitative ^99m^Tc-PYP SPECT/CT for therapy monitoring in ATTR-CA. Volumetric indices (AmyDV, TAU) demonstrated marked reductions with tafamidis, suggesting sensitivity to treatment-related changes beyond visual or semi-quantitative indices. Matsuda et al. recently reported the utility of these indices in PYP and ^67^Ga-citrate SPECT/CT, establishing methodological validation (6, 7).

 An important observation was the discordance between imaging and clinical/functional outcomes. Despite imaging improvement, the patient’s symptoms persisted, GLS worsened, and BNP rose. In addition, left ventricular wall thickness also remained increased, consistent with the notion that tafamidis prevents further amyloid deposition but does not reverse pre-existing hypertrophy. Tafamidis stabilizes transthyretin tetramers to prevent new fibril formation, but does not promote clearance of existing deposits. Decreased tracer uptake may reflect altered binding dynamics or partial remodeling rather than true regression of amyloid (8). Fibrosis or other structural changes could also influence tracer retention. This emphasizes cautious interpretation of imaging biomarkers.

 In particular, the use of volumetric indices in this case demonstrates how recent advances in SPECT/CT quantification may complement established visual and semi-quantitative methods. These approaches, originally developed in oncological PET, have now been adapted to cardiac imaging, and our experience adds to this evolving field. Several reports have indicated that imaging improvement does not always parallel symptomatic or biomarker improvement, underlining the complexity of ATTR-CA and the need for multiparametric follow-up. Larger multicenter studies are warranted to clarify whether quantitative indices such as AmyDV and TAU can serve as surrogate endpoints in clinical trials and guide therapeutic decisions in routine practice.

 Integration with multimodality imaging is also essential. Echocardiography provides functional assessment, while biomarkers reflect hemo-dynamic stress. Cardiac MRI with T1 mapping could distinguish amyloid from fibrosis (9). 

 Future studies must determine whether volumetric PYP indices predict survival or hospitalization, and establish validated thresholds. Reports by Lee et al. (2023) (10) and Okada et al. (2023) (11), as well as Yu et al. (2025) (12), demonstrated reductions in PYP uptake during tafamidis therapy. More recently, 

Yu et al. (2024) (13) extended these findings to patients receiving eplontersen, further supporting the role of quantitative PYP SPECT/CT in therapy monitoring. Vijayakumar et al. showed quantitative changes in patients on stabilization therapy (14). Our case further supports these findings, providing evidence that volumetric indices may detect therapy-related changes, though they do not necessarily parallel functional recovery.

## Conclusion

 Quantitative ^99m^Tc-PYP SPECT/CT demonstrated marked reductions in tracer uptake following tafamidis therapy in wild-type ATTR-CA. Novel volumetric indices such as AmyDV and TAU may serve as promising biomarkers for treatment monitoring; however, their interpretation should be integrated with clinical status, echocardiographic findings, and biomarkers. Larger prospective studies are warranted to validate these indices and clarify their prognostic significance.
